# Protein C Deficiency Caused by a Novel Mutation in the* PROC* Gene in an Infant with Delayed Onset Purpura Fulminans

**DOI:** 10.1155/2017/8915608

**Published:** 2017-09-26

**Authors:** Mariam S. Al Harbi, Ayman W. El-Hattab

**Affiliations:** ^1^Department of Pediatrics, Tawam Hospital, P.O. Box 15258, Al-Ain, UAE; ^2^Division of Clinical Genetic and Metabolic Disorders, Tawam Hospital, P.O. Box 15258, Al-Ain, UAE

## Abstract

Protein C is an anticoagulant that is encoded by the* PROC* gene. Protein C deficiency (PCD) is inherited in an autosomal dominant or recessive pattern. Autosomal dominant PCD is caused by monoallelic mutations in* PROC* and often presents with venous thromboembolism. On the other hand, biallelic* PROC *mutations lead to autosomal recessive PCD which is a more severe disease that typically presents in neonates as purpura fulminans. In this report, we describe an 8-month-old infant with autosomal recessive PCD who presented with multiple lumps on his lower extremities at the age of 2 months and later developed purpura fulminans after obtaining a muscle biopsy from the thigh at the age of 5 months. Protein C level was less than 10% and* PROC* gene sequencing identified a novel homozygous missense mutation, c.1198G>A (p.Gly400Ser). Autosomal recessive PCD typically presents with neonatal purpura fulminans which is often fatal if not recognized and treated early. Therefore, early recognition is critical in preventing morbidity and mortality associated with autosomal recessive PCD.

## 1. Introduction

Protein C, a vitamin K-dependent factor synthesized in the liver, plays a significant role in regulating the coagulation cascade. It circulates as a zymogen and exerts its anticoagulant function after being activated on endothelial surfaces following binding to the endothelial protein C receptor. The inhibitory effect of activated protein C is enhanced by protein S. Protein C primarily inactivates factors V and VIII, hence preventing thrombin generation and thrombosis formation.* PROC*, the gene encoding protein C, is located on chromosome 2q14.3 and contains 9 exons [1].

Similar to other inherited thrombophilias, a deficiency in protein C can predispose to thrombosis. Protein C deficiency (PCD) can be classified into type I, where there is a decrease in protein C concentration, and type II, where there is a decreased activity with normal level of protein C. Type I deficiency results from defective synthesis or secretion of the protein, whereas type II results from impaired binding to substrate, calcium, or receptor. Type I deficiency is the most common type, whereas type II accounts for 10–15% of cases [2].

PCD can be an autosomal dominant or recessive disease. Autosomal dominant PCD is caused by heterozygous (monoallelic) mutations in* PROC* and has an incidence of 1 in 200–500. Individuals with this form have a plasma protein C level around 50% of normal values. Affected individuals with autosomal dominant PCD are typically asymptomatic; however, some may develop venous thromboembolism during childhood or later as young adults [1]. Biallelic (homozygous or compound heterozygous)* PROC *mutations lead to the autosomal recessive PCD which occurs in 1 in 40,000–250,000 individuals. Autosomal recessive PCD, which is more severe than autosomal dominant PCD, is associated with very low level of protein C and typically presents in neonatal period with neonatal purpura fulminans [3].

Herein, we describe an infant who presented with delayed onset purpura fulminans and was found to have autosomal recessive PCD caused by a novel homozygous* PROC* mutation.

## 2. Case Presentation

**Figure 1:**
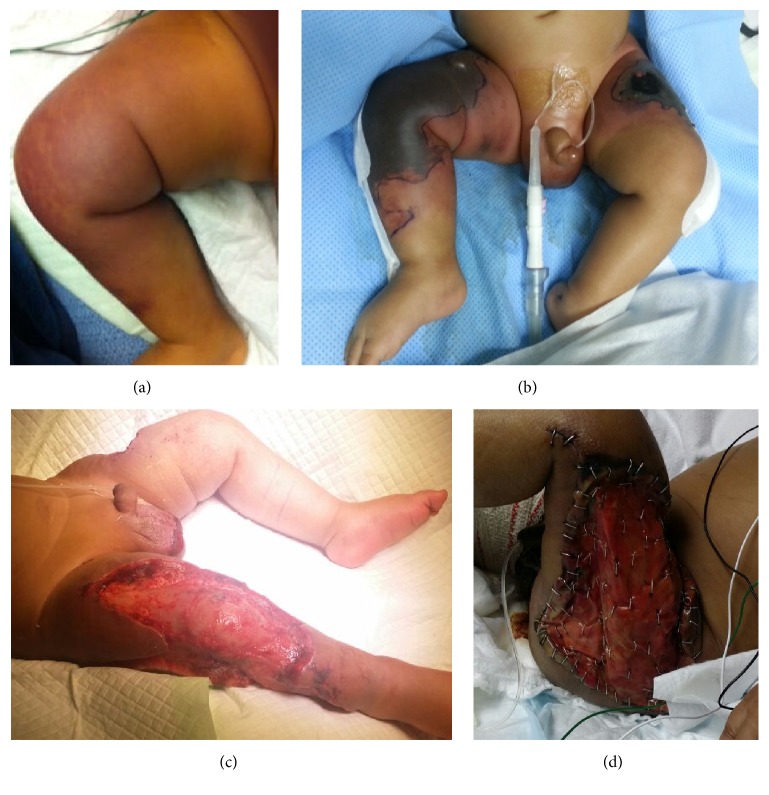
Skin changes at various stages: (a) on the 6th day of hospitalization showing discoloration after the muscle biopsy; (b) on the 8th day of hospitalization showing hemorrhagic necrosis; (c) on the 12th day of hospitalization after fasciotomy and debridement; (d) on the 12th day showing Integra application.

An 8-month-old male infant developed a perianal mass during the first week of life which was treated as an abscess. The infant at that time had a full septic work-up that was negative and it was decided to treat him conservatively with antibiotic without incision and drainage. At the age of 2 months, he started having recurrent subcutaneous lumps on the lower extremities. At 5 months of age, he was provisionally diagnosed to have panniculitis and was hospitalized at that time for a muscle biopsy from the right thigh to confirm the diagnosis of panniculitis and evaluate other possible etiologies. After the muscle biopsy, he developed rapidly progressive bluish discolorations and indurations in the upper thighs which extended to the buttocks and scrotum over a period of 2 days (Figure 1(a)). The skin lesions progressed to hemorrhagic necrosis on the 8th day of hospitalization (Figure 1(b)). When the hemorrhagic necrosis developed, purpura fulminans was considered and protein C deficiency was suspected. He was started on regular fresh frozen plasma transfusion and later started on protein C concentrate and low-molecular-weight heparin. His course was complicated by compartment syndrome that required bilateral lower extremity fasciotomy followed by extensive debridement with artificial dermis (Integra®) application (Figures 1(c) and 1(d)). Additionally, he developed anemia requiring multiple blood transfusions, thrombocytopenia, consumptive coagulopathy (prolonged prothrombin time (PT), activated partial thromboplastin time (aPTT), and low fibrinogen), and fibrinolysis (increased D-dimer) which were consistent with disseminated intravascular coagulation (DIC). His illness was further complicated by Gram-negative sepsis* (Klebsiella oxytoca* and* Enterobacter cloacae)*, hypertension, and respiratory failure requiring ventilatory support. The infant was hospitalized for 3 months during which he gradually improved while receiving protein C concentrate. He was transferred to another facility for further care and skin grafting at the age of 8 months.

His growth parameters were appropriate for the age. His parents were cousins and they had 6 other older children who were reportedly healthy and there was no family history of any hematological diseases.

Protein C level was found to be very low (less than 10%, normal: 68–143%) consistent with autosomal recessive PCD. The* PROC* gene was sequenced at PreventionGenetics LLC, Marshfield, Wisconsin, USA, and a novel homozygous missense mutation (c.1198G>A; p.Gly400Ser) was identified, confirming the diagnosis of autosomal recessive PCD. Protein C level was 56% in the mother and 73% in the father (normal: 68–143%), suggesting that both parents are carriers and have autosomal dominant PCD. Genetic testing showed that both parents are heterozygous for the p.Gly400Ser mutation in* PROC*, confirming the diagnosis of autosomal dominant PCD in both parents. Both parents were healthy with no history suggestive of thrombophilia.

## 3. Discussion

Purpura fulminans describes microvascular thrombosis associated with DIC and perivascular hemorrhage. It usually presents with cutaneous purpuric lesions that start as dark-red and then become purple-black indurated lesions. It occurs at sites of previous traumas like intravenous cannula sites. There is a predilection to limbs but the lesions can also occur on the buttocks and thighs. With time, these lesions become gangrenous and can result in the loss of extremities. There are both congenital and acquired causes of purpura fulminans. Inherited causes are due to an autosomal recessive PCD or protein S deficiency [4]. Acquired causes are more common and are often associated with severe infections causing a consumptive coagulopathy and a relative deficiency of protein C and/or S. The clinical severity may vary depending on the underlying cause; however, the condition is often fatal if not recognized and treated early. The onset of symptoms in severe congenital causes is usually within the first days of life [5].

The suspicion of PCD in the infant presented here appeared after the infant developed severe hemorrhagic necrotic skin lesions and DIC consistent with purpura fulminans. The skin lesions in purpura fulminans usually start after a trauma. In the infant described here, the muscle biopsy was the trauma that precipitated the development of purpura fulminans. Although purpura fulminans due to severe congenital causes usually occurs within the first few days of life, infants presenting with a delayed onset purpura fulminans, between 6 and 12 months of age, were reported [6, 7]. The infant in this report had a delayed purpura fulminans which appeared at the age of 5 months. Purpura fulminans is often fatal if not recognized and treated early [5]. The clinical presentation of hemorrhagic skin necrosis and DIC in the infant reported here raised the suspicion of this condition and treatment was immediately started. Early recognition is critical in preventing morbidity and mortality associated with this disease.

Individuals with autosomal recessive PCD carry biallelic* PROC *mutations and have very low level of protein C, whereas individuals carrying heterozygous* PROC *mutations have protein C level at about 50% of reference values [1]. The infant in this report had very low protein C level consistent with autosomal recessive PCD and his parents exhibited low protein C levels consistent with heterozygous carrier status. Molecular studies have further confirmed the diagnosis as the affected infant was found to have a homozygous mutation in the* PROC* gene and both parents were found to be heterozygous for this mutation. The identified* PROC* novel mutation c.1198G>A (p.Gly400Ser) was not previously reported. This mutation is located in a highly conserved region of the enzyme and the amino acid substitution prediction programs (PolyPhen-2, SIFT, and MutationTaster) predicted that this variant is deleterious. These* in silico* data along with the biochemical and clinical phenotypes support that p.Gly400Ser is indeed a disease causing mutation that is responsible for PCD in this infant.

In conclusion, the classic presentation of autosomal recessive PCD is neonatal purpura fulminans. Early recognition is critical in preventing morbidity and mortality associated with autosomal recessive PCD. Although the identified p.Gly400Ser mutation in* PROC* was not previously reported, its pathogenicity is supported by different* in silico* prediction programs and the biochemical and clinical phenotypes that are consistent with autosomal recessive PCD. Therefore, p.Gly400Ser in* PROC* is a novel mutation causing autosomal recessive PCD that presented in this infant with late onset purpura fulminans.
